# High‐Dose Phenprocoumon Intoxication Treated With Therapeutic Plasma Exchange

**DOI:** 10.1002/jca.70062

**Published:** 2025-10-02

**Authors:** Lea U. Krauß, Andreas M. Brosig, Patricia Mester, Tanja Elger, Stephan Schmid, Martina Müller, Vlad Pavel

**Affiliations:** ^1^ Department of Internal Medicine I, Gastroenterology, Hepatology, Endocrinology, Rheumatology, and Infectious Diseases University Hospital Regensburg Regensburg Germany; ^2^ Institute of Clinical Chemistry and Laboratory Medicine, Transfusion Medicine University Hospital Regensburg Regensburg Germany

## 1 Background

Phenprocoumon inhibits the synthesis of coagulation factors II, VII, IX, and X, as well as proteins C and S [1]. It is commonly used for prophylactic anticoagulation, particularly after artificial heart valve replacement. Therapeutic monitoring is performed using the International Normalized Ratio (INR) [2], with a therapeutic plasma level of phenprocoumon (PLP) ranging between 1.0 and 3.5 mg/L. Overdoses are typically due to poor compliance; however, cases involving suicidal intent have also been described [3]. Overdose may lead to gastrointestinal bleeding, cerebral bleeding, or liver injury [4, 5]. In massive overdoses, dialysis is ineffective because of the high plasma protein binding of the drug [6].

This work was reviewed and approved by the Ethics Committee of the University of Regensburg, Regensburg, Germany (25‐4254‐104). Written informed consent for publication was obtained from the patient.

## 2 Case Report

A 78‐year‐old woman presented to the emergency department 13 h after the intentional ingestion of 300 mg phenprocoumon. She denied any bleeding, gastrointestinal, or neurological symptoms. Her medical history included atrial fibrillation and mechanical heart valve replacement. The target INR was between two and three. Given the high risk of bleeding, she was admitted to the intensive care unit (ICU).

Initial laboratory tests revealed an INR of 2.9, a partial thromboplastin time (PTT) of 34.3 s, and a PLP of 51.4 mg/L. However, it is known that the anticoagulant effect of phenprocoumon starts with a latency of approximately 48–72 h [3]. Analysis of coagulation factors revealed abnormal values of factor VII (38%) and factor IX (47%). The coagulometric method was performed for measuring INR. Thromborel S was used as a reagent. After an initial dose of vitamin K1, the PLP remained high at 48.5 mg/L. Due to the drug's > 98% plasma protein binding and long half‐life [5], we opted for therapeutic plasma exchange (TPE) to facilitate the elimination of albumin‐bound phenprocoumon.

Since ultrasound of the neck vessels revealed optimal anatomy for a safe puncture, a central line catheter was placed in the right internal jugular vein. If the placement of a central line is considered high‐risk for bleeding complications, in most cases TPE can be performed also using a peripheral catheter [7]. Daily TPE using fresh frozen plasma (FFP) was performed for three consecutive days using the Spectra Optia cell separator (Terumo BCT Inc., Lakewood, CO). Each TPE involved 1.3 times the patient's plasma volume (averaging 48.9 mL/kg body weight) (Table 1).

**TABLE 1:** Data of therapeutic plasma exchange.

Plasmapheresis	Replacement fluid used (ml)	Removed plasma (ml)	Exchanged plasma volume (x‐fold)	Inlet flow (ml/min)	Duration of the procedure (min)	Exchanged plasma volume (ml/kg)
1	2448	2557	1.3	48.73	107	49
2	2427	2553	1.3	49.69	115	48.5
3	2454	2583	1.3	45.22	123	49.1
Average value	2443	2564.3	1.3	47.9	115	48.9

Due to the presence of mechanical heart valves, PTT‐controlled anticoagulation with unfractionated heparin began 15 h after ingestion. Vitamin K1 was administered repeatedly, guided by INR and PTT values.

**FIGURE 1:**
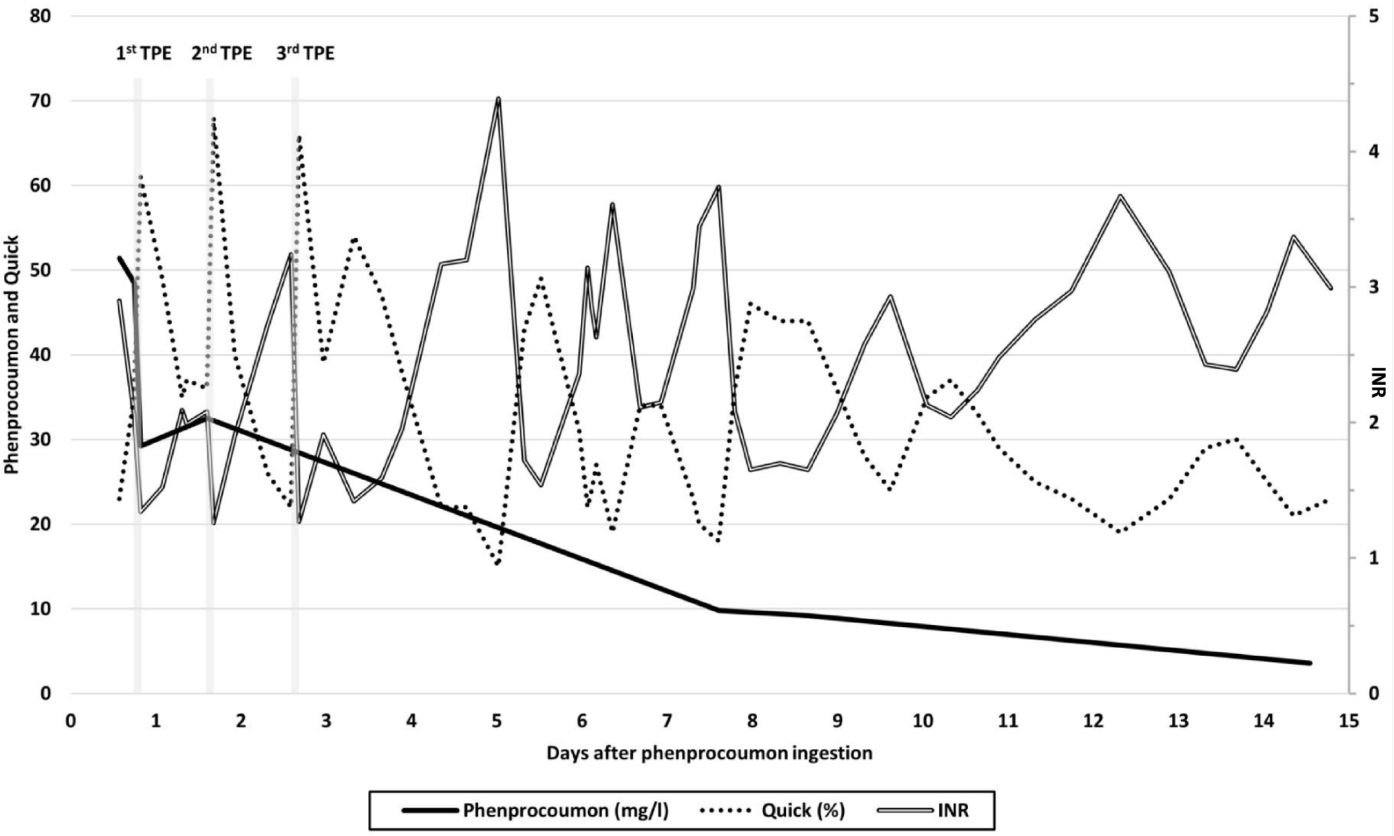
Serum levels of phenprocoumon, quick time, and INR. *TPE = therapeutic plasma exchange.

After the first TPE, PLP dropped to 29.2 mg/L. 14 days post‐ingestion, the level declined to 3.6 mg/L, which is below the toxic threshold (Figure 1). Post‐TPE variability of INR and Quick time values may be explained by the various individual absorption rates from the gastrointestinal tract that, in some cases, can take longer, and by the long elimination half‐life of phenprocoumon. Furthermore, it is known that phenprocoumon needs a longer time, almost 2 weeks, to reach stable blood values [6, 8]. The ICU course was uneventful aside from a transient rise in liver enzymes and a short episode of macrohematuria, both resolving spontaneously. Following stabilization, the patient was transferred to psychiatric care.

## 3 Discussion

Standard treatment for phenprocoumon overdose includes vitamin K1 administration. In addition, prothrombin complex concentrate (PCC), FFP, or cholestyramine may be employed [1, 9]. However, patients with mechanical heart valves have an increased risk for thrombosis [10]. Only one prior case has reported successful management of a life‐threatening phenprocoumon overdose with TPE [9]. In that case, a patient ingested 330 mg of phenprocoumon, presenting with a PLP of 7.4 mg/L and significant bleeding. Despite conventional therapy—including plasma, erythrocyte transfusions, PCC, vitamin K1, and cholestyramine—no improvement was noted until plasmapheresis was initiated. Two sessions reduced the serum level from 4.0 to 0.9 mg/L, and the patient stabilized rapidly, with discharge occurring on hospital day 20. The reported toxin elimination was 77.5% after two TPE [9].

In contrast, our patient had a substantially higher initial PLP of 51.4 mg/L. Given the elevated bleeding risk, we initiated plasmapheresis early, achieving a 39.8% toxin reduction after the first session. Early and decisive intervention likely prevented serious hemorrhagic complications.

Both cases illustrate the utility of plasmapheresis for rapidly decreasing toxic phenprocoumon levels. Although further data are needed, plasmapheresis may offer a life‐saving treatment alternative when traditional therapies are insufficient. Clinicians should consider this modality, especially in cases of massive overdose with high PLP and bleeding risk.

## Conflicts of Interest

The authors declare no conflicts of interest.

## Data Availability

The data that support the findings of this study are available on request from the corresponding author. The data are not publicly available due to privacy or ethical restrictions.
